# Roles of PCNA ubiquitination and TLS polymerases κ and η in the bypass of methyl methanesulfonate-induced DNA damage

**DOI:** 10.1093/nar/gku1301

**Published:** 2014-12-10

**Authors:** Niek Wit, Olimpia Alessandra Buoninfante, Paul C.M. van den Berk, Jacob G. Jansen, Marc A. Hogenbirk, Niels de Wind, Heinz Jacobs

**Affiliations:** 1 Division of Biological Stress Responses, The Netherlands Cancer Institute, Amsterdam, The Netherlands; 2 Department of Human Genetics, Leiden University Medical Center, Leiden, The Netherlands

## Abstract

Translesion synthesis (TLS) provides a highly conserved mechanism that enables DNA synthesis on a damaged template. TLS is performed by specialized DNA polymerases of which polymerase (Pol) κ is important for the cellular response to DNA damage induced by benzo[a]pyrene-7,8-dihydrodiol-9,10-epoxide (BPDE), ultraviolet (UV) light and the alkylating agent methyl methanesulfonate (MMS). As TLS polymerases are intrinsically error-prone, tight regulation of their activity is required. One level of control is provided by ubiquitination of the homotrimeric DNA clamp PCNA at lysine residue 164 (PCNA-Ub). We here show that Polκ can function independently of PCNA modification and that Polη can function as a backup during TLS of MMS-induced lesions. Compared to cell lines deficient for PCNA modification (*Pcna*^K164R^) or Polκ, double mutant cell lines display hypersensitivity to MMS but not to BPDE or UV-C. Double mutant cells also displayed delayed post-replicative TLS, accumulate higher levels of replication stress and delayed S-phase progression. Furthermore, we show that Polη and Polκ are redundant in the DNA damage bypass of MMS-induced DNA damage. Taken together, we provide evidence for PCNA-Ub-independent activation of Polκ and establish Polη as an important backup polymerase in the absence of Polκ in response to MMS-induced DNA damage.

## INTRODUCTION

Translesion synthesis (TLS) is an evolutionary conserved DNA damage tolerance (DDT) pathway that enables cells to cope with replication-blocking DNA lesions (1). TLS allows continuation of DNA replication on damaged templates, thereby preventing replication fork collapse that may trigger cell death or genome instability. TLS depends on specialized DNA polymerases that are able to accommodate bulky DNA lesions as well as non-Watson–Crick base paring in their flexible active sites (2). Moreover, these enzymes lack the 3′ to 5′ exonuclease activity associated with the proofreading ability of replicative polymerases. Together, these characteristics render TLS polymerases intrinsically error-prone in replicating across DNA lesions. It was previously thought that TLS occurred via a late, post-replicative mechanism (3–6). Indeed, genetic studies in yeast showed that functional TLS can occur in the G2 phase of the cell cycle, thus uncoupled from replication (7,8). However, recent evidence suggested that, depending on the polymerase and the lesion, TLS could also occur directly at the replication fork, also called ‘on the fly’ (9,10).

Polκ belongs to the Y-family of TLS polymerases and is capable of both incorporating nucleotides opposite certain DNA lesions and extending from mismatched primer termini (1). *In vitro* Polκ is able to incorporate deoxycytidinemonophosphate (dCMP) opposite different *N^2^*-linked dG adducts, including *N^2^*-furfuryl-dG, *N^2^*-(1-carboxyethyl)-2-dG and *N^2^*-BPDE-dG adducts (11–16). Additionally, the cellular response to DNA damage induced by environmental carcinogens BPDE, ultraviolet (UV) light or by the monofunctional alkylating agent methyl methanesulfonate (MMS) revealed a critical role for Polκ (17–20). Consistently, Polκ-deficient mammalian cells are sensitive to and hypermutable for BPDE (20,21). Mice lacking Polκ display a spontaneous mutator phenotype in various tissues and a reduced survival rate compared to wild-type (WT) mice (22,23). Due to similar mutation spectra of Polκ-deficient and BPDE-treated mice, it was suggested that the primary *in vivo* role of Polκ is to prevent mutagenesis by naturally occurring polycyclic aromatic hydrocarbon guanine adducts (22).

The role of Polκ in TLS requires strict regulation of its activity. Polκ contains five domains that could be important for its regulation: a domain that mediates interaction with the TLS polymerase and TLS regulator Rev1, a PCNA-interacting protein (PIP) domain, two ubiquitin-binding zinc finger domains and a nuclear localization domain (1,24,25). However, the mechanism by which Polκ is activated at stalled replication complexes is still unclear. A central player in controlling TLS polymerase activity is thought to be the homotrimeric DNA clamp PCNA, which normally acts as a processivity factor for replicative DNA polymerases (26,27). Upon stalling of the replication machinery at a DNA lesion, PCNA is subjected to monoubiquitination at K164 by the ubiquitin conjugase/ligase dimer Rad6/18 (PCNA-Ub) (28). This event may initiate a polymerase switch from a replicative polymerase to a TLS polymerase by (i) preventing the binding of a set of proteins that would otherwise inhibit the binding of TLS polymerases to PCNA (29,30) or (ii) by increasing the affinity of TLS polymerases for binding to PCNA via their ubiquitin binding domain(s) (UBD) (31–35). Indeed, mammalian Y-family TLS polymerases η, ι and Rev1 display a higher affinity for PCNA-Ub than for unmodified PCNA (31,32). TLS can occur independently of PCNA-Ub (36,37). The relevance of PCNA-Ub in the activation of Polκ, however, is less clear, as some studies report that, for example, Polκ critically needs its UBD for activation (38), while others have reported that Polκ can participate in RAD18-independent TLS of UV lesions (39).

To determine the requirement of PCNA-Ub in the activation of Polκ in a genetically well-defined mammalian system, we here established homozygous *Pcna^K164R^;Polk^−/−^*, as well as WT and single mutant cell lines. We report that Polκ can be activated in the absence of PCNA-Ub in response to MMS-induced DNA damage, as is shown by more severe defects in cell survival, replication fork progression, cell cycle progression and higher levels of replication stress in *Pcna^K164R^;Polk^−/-^* cells compared to the respective single mutant cells. Additionally, we established Polη as an important back-up polymerase for TLS past MMS-induced lesions, as *Polh^−/−^* cells do not display any sensitivity to MMS or defects in replication fork progression, *Polk^−/−^;Polh^−/−^* cells do display strong phenotypes, at even higher levels than *Polk^−/−^* cells.

## MATERIALS AND METHODS

### Primary cell isolation and cell culture

The generation and genotyping of *Pcna^K164R^* and *Polk^−/−^* mice have been described elsewhere (17,40). Primary mouse embryonic fibroblasts (MEFs) and fetal livers were isolated from E14.5 embryos derived from intercrosses of heterozygous *Pcna^K164R^*; *Polk^+/−^* mice. Pre-B cells were generated from single cell suspensions of fetal livers grown on lethally γ-irradiated ST2 cells in complete medium (IMDM, supplemented with 8% fetal calf serum (FCS), pen/strep and β-mercaptoethanol) containing IL-7, according to (41). Primary MEFs were cultured under low (3%) oxygen tension, with 5% CO_2_ at 37°C. To immortalize MEFs, primary MEF cultures were transduced with a lentivirus encoding a p53-specific shRNA (42).

### Cell survival

For UV-C treatment, 10^5^ pre-B cells were seeded in 24-wells plates containing an ST2 feeder layer and 0.5-ml complete medium and IL-7. After 15 min cells were irradiated (254 nm, UV Stratalinker 2400) and cultured in total 1 ml complete medium and IL-7. For cisplatin, MMS, BPDE, H_2_O_2_ and γ-irradiation treatment 10^5^ pre-B cells were seeded in 24-wells plates containing an ST2 feeder layer and 1 ml complete medium and IL-7 in the continuous presence of different doses of the above-mentioned compounds or after different single doses of γ irradiation from a ^137^Cs source. To determine cell survival, cells were harvested after 3 days of culture and stained with propidium iodine (PI). The number of PI-negative cells was measured on a FACSArray (Becton Dickinson). Data analysis was performed with FlowJo software.

### MMS-induced foci formation

The generation of WT and *Pcna^K164R^* MEFs containing eGFP-Polκ is described elsewhere (37). Per well 1.5 × 10^5^ MEFs were seeded on a glass coverslip in 6-well plate. One day later, complete medium was added with or without 0.75-mM MMS. The cells were incubated for 6 h in the presence of MMS, after which the cells were washed with phosphate buffered saline (PBS) containing Ca^2+^ and Mg^2+^ and fixed in 4% paraformaldehyde in PBS containing Ca^2+^ and Mg^2+^ for 5 min. Cells were washed with PBS containing Ca^2+^ and Mg^2+^, after which the coverslips were mounted in Fluoro-Gel (Electron Microscopy Sciences). Microscopy was performed using a fluorescent microscope (Zeiss). At least 250 cells were analyzed per genotype.

### DNA fiber analysis

Per well 7.5 × 10^4^ MEFs were seeded in a 6-well plate in 3-ml complete medium. The next day the medium was removed and 1-ml complete medium was added. Next, 1 ml complete medium containing 50-μM 5-Chloro-2′-deoxyuridine (CldU) was added. Exactly 20 min later 2 ml complete medium containing 500 μM 5-Iodo-2′-deoxyuridine (IdU) and ±6 mM MMS was added. Exactly 20 min later the cells were washed with PBS containing Ca^2+^ and Mg^2+^, trypsinized and counted. For each condition a cell suspension of 3.0 × 10^5^ cells/ml was prepared. Two microliter of this cell suspension was added to a microscope slide (Menzel-Gläser Superfrost, Fisher Scientific) and air-dried for 5 min. The cells were lysed by adding 7 μl lysis buffer (200-mM Tris-HCl pH7.4, 50-mM ethylenediaminetetraacetic acid, 0.5% sodium dodecyl sulphate (SDS)) and swirled vigorously with a pipet tip. After air-drying for 3 min the slides were raised to an angle of 15° and the remaining drop was allowed a minimum of 2 min to run down the bottom of the slide after which the slide was air-dried completely. Fixation was performed with 3:1 methanol:acetic acid for 10 min. After the slides were air-dried completely, they were stored at 4°C. For immunostaining, the slides were rehydrated two times in dH_2_O, washed once in 2.5 M HCl and denatured for 75 min in 2.5 M HCl. The slides were then washed twice with PBS and twice with blocking solution (PBS containing 1% bovine serum albumin (BSA) and 0.1% Tween-20). Blocking was performed by incubating the slides for 50 min in blocking solution. Rat-anti-BrdU antibody (BU1/75, AbD Serotec) 1:500 diluted in blocking solution and Mouse-anti-BrdU (Clone B44, BD) 1:750 diluted in blocking solution were added for exactly 60 min to detect incorporated CldU and IdU, respectively. Subsequently, the slides were washed three times with PBS and fixed using 4% paraformaldehyde in PBS for 10 min, after which the slides were washed three times in PBS and three times in blocking solution. Secondary antibodies (Goat-anti-Rat Alexa Fluor-555 (Molecular Probes) and Goat-anti-Mouse Alexa Fluor-488 (Molecular Probes)), both 1:500 diluted in blocking solution, were then added for 90 min in the dark. The slides were then washed twice in PBS and three times in blocking solution in the dark. Finally, slides were mounted in Fluoro-Gel (Electron Microscopy Sciences) and stored in the dark at 4°C (43). Microscopy was performed using a fluorescent microscope (Zeiss).

### Alkaline DNA unwinding

Per well 5 × 10^4^ MEFs were seeded in a 24-well plate and cultured overnight. Cells were either MMS (1.5 mM in serum-free medium) or mock treated for 30 min. MEFs were pulse labeled with [^3^H]thymidine (2 μCi/ml; 76 Ci/mmol) for 30 min and cultured in medium for up to 6 h. At different time points, the cells were washed twice with 0.15 M NaCl and incubated in the dark on ice in 0.5 ml of ice-cold unwinding solution containing 0.15 M NaCl and 0.03 M NaOH for 30 min. Unwinding was terminated by forceful injection of 1 ml of 0.02 M NaH_2_PO_4_. The cell lysates were sonicated for 30 s using a Sonifier 250 apparatus (Branson); SDS was added up to 0.25% and the plates were stored at −20°C. To separate single-stranded DNA (ssDNA) from double-stranded DNA (dsDNA), hydroxyl apatite columns were washed with 0.5 M K_2_HPO_4_ followed by 10 mM NaH_2_PO_4_ (pH 6.8). After each cell lysate was loaded, the columns were washed twice with 10 mM NaH_2_PO_4_ (pH 6.8). ssDNA was eluted with 0.1 M K_2_HPO_4_ (pH 6.8), and double-stranded DNA was eluted with 0.3 M K_2_HPO_4_ (pH 6.8). Radioactivity was quantified by liquid scintillation counting (10).

### Cell cycle analysis and γH2A.X FACS staining

Per condition 2 × 10^5^ pre-B cells were exposed in 1-ml complete medium to 100 μM MMS for 30 min at 37°C. At indicated time points cells were harvested and fixed immediately with 3 ml ice-cold 100% ethanol and kept at 4ºC. Cells were treated for 20 min with RNAse A (0.5 mg/ml, Sigma Chemical Co.), before permeabilizing in Tween-20 solution (0.25% Tween-20 in PBS/1% BSA). γH2A.X was detected with 1-μg/ml anti-phospho-histone H2A.X antibody (Ser139, clone JBW301; Millipore). After staining with fluorescein isothiocyanate-conjugated anti-mouse IgG antibody (25 μg/ml) (DAKO Cytomation), the cells were resuspended in PBS containing 0.5 μM TO-PRO®-3 (Invitrogen) and measured on a FACSCalibur (Becton Dickinson). Data were analyzed using FlowJo software.

### Sample preparation and immunoblotting

To detect pCHK1 S345, 7.5 × 10^5^ MEFs were seeded in a 10 cm dish per condition. One day later, the cells were mock or pulse treated with 5 mM MMS in complete medium for 30 min and harvested at the indicated time points by scraping on ice. The cell pellet was snap frozen in liquid nitrogen and stored at −80°C. For whole cell extract preparation, cell pellets were thawed on ice and lysed in 50-μl RIPA buffer (25 mM TrisHCl (pH 7.6), 150 mM NaCl, 1% NP-40, 1% sodium deoxycholate and 0.1% SDS) containing 1 mM phenylmethylsulfonyl fluoride (PMSF), 1x protease inhibitor cocktail (Roche), 1x PhosStop (Roche), and incubated for 30′ on ice, sonicated for 10 min and centrifuged for 10 min at 20 800 x g (4°C). Protein concentration in the supernatant was measured using the Bradford method. Immunoblotting was performed according to standard protocols. NuPAGE 4–12% gels (Invitrogen) were used for protein separation. Antibodies used were: rabbit anti-pChk1 S345, 1:1000 (clone 133D3, Cell Signaling); mouse anti-Actin, 1:10 000 (clone C4 (MAB1501R), Millipore), goat anti-rabbit-IRDye 800CW (Licor) and goat anti-mouse-IRDye 680RD (Licor).

For PCNA-Ub detection after MMS treatment, 1.5 × 10^6^ cells were seeded on a 15-cm dish per condition. One day later cells were mock treated or treated continuously with 0.75-mM MMS in complete medium. After 6 h, cells were washed in cold PBS containing 2 mM *N*-ethylmaleimide (NEM), scraped on ice, snap frozen in liquid nitrogen and stored at −80°C. To isolate nuclei, cells were thawed on ice and lysed in buffer A (50 mM HEPES pH 6.8, 100 mM NaCl, 300 mM sucrose, 3 mM MgCl_2_, 1 mM EGTA pH 8.0, 0.2% Triton X-100, 1x protease inhibitors cocktail (freshly added, Roche), 2 mM NEM (freshly added), 1 mM PMSF (freshly added)) for 10 min. Nuclei were centrifuged at 510 x g at 4°C for 10 min and washed in buffer A. Subsequently, nuclei were lysed in 0.1% SDS buffer (50 mM Tris pH 7.5, 150 mM NaCl, 0.1% SDS), and the lysate was sonicated and centrifuged (20,800 x g at 4°C for 10 min) to obtain chromatin proteins (supernatant). Western blotting was performed using standard protocols. NuPAGE 12% gels (Invitrogen) were used for protein separation. Antibody used was: mouse anti-PCNA-HRP (PC-10, Santa Cruz).

### Colony survival

*Polh*^−/−^; *Polk*^−/−^, single mutant and wild-type MEFs were isolated from embryos from *Polh*^+/−^ x *Polk*^+/−^ crosses described elsewhere (44). MEFs were seeded in 10 cm dishes in complete medium with varying cell concentrations. One day later, the medium was removed and replaced with complete medium containing the indicated concentrations of MMS. Five to eight days later the cells were washed with PBS and fixed in 5 ml of methanol:acetic acid (3:1) for 1 h. Colonies were stained by adding 3 ml 0.3% Coomassie Brilliant Blue in H_2_O. After 1 h, the staining solution was removed and the dished were washed with H_2_O and allowed to dry. Survival of MMS-treated cells was corrected for the plating efficiency of the untreated cells. Data points represent the mean survival relative to the untreated control cells.

## RESULTS

### Sensitivity of WT, Pcna^K164R^, Polk^−/−^ and Pcna^K164R^; Polk^−/−^ cells to different DNA damaging agents

**Figure 1.**
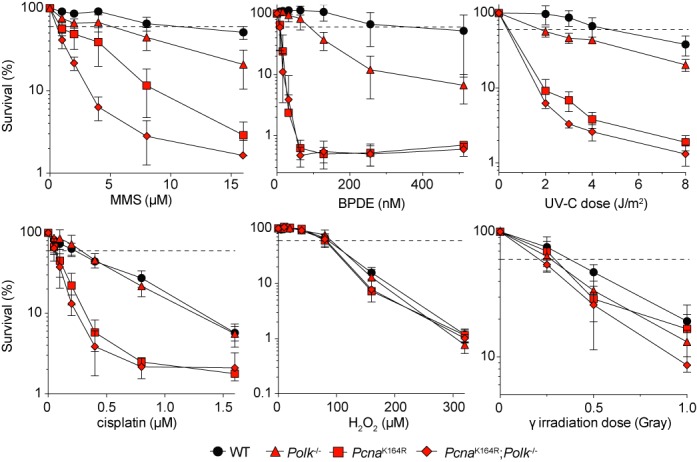
Cell survival in response to different DNA damaging agents of *Pcna*^K164R^; *Polk*^−/−^ pre-B cells. Pre-B cell survival was normalized to the mock-treated cells for each condition. The average of two independent experiments with two independent cell lines per genotype *in duplo* is plotted ±SD. The dashed line indicates 50% survival.

To investigate the requirement of PCNA-Ub in the activation of Polκ, we intercrossed heterozygous *Pcna^K164R^*; *Polk^+/−^* mice to generate WT, the respective single mutants and double mutant embryos. Thereof, primary pre-B cells were assessed for their sensitivity to a panel of DNA damaging agents (Figure 1). No significant differential sensitivity was found in all four genotypes when these cells were exposed to either H_2_O_2_ or γ-irradiation. While *Pcna*^K164R^ cells were hypersensitive to the crosslinking agent cisplatin, Polκ-deficient cells were not. In line with previous studies, Polκ-deficient pre-B cells were sensitive to MMS (19), BPDE (20) and UV irradiation (17) (Figure 1). For these three tested genotoxins the *Pcna*^K164R^ mutant cells displayed a higher sensitivity than the Polκ-deficient cells, emphasizing the importance of PCNA-Ub in the DNA damage response, possibly by controlling TLS polymerase activity. Remarkably, *Pcna^K164R^;Polk^−/−^* cells are even more sensitive to MMS than the *Pcna*^K164R^ single mutant, whereas this is not observed for BPDE and UV. Our data indicate the existence of a lesion-specific, PCNA-Ub-independent pathway for the activation of Polκ.

### MMS-induced eGFP-Polκ foci formation

**Figure 2.**
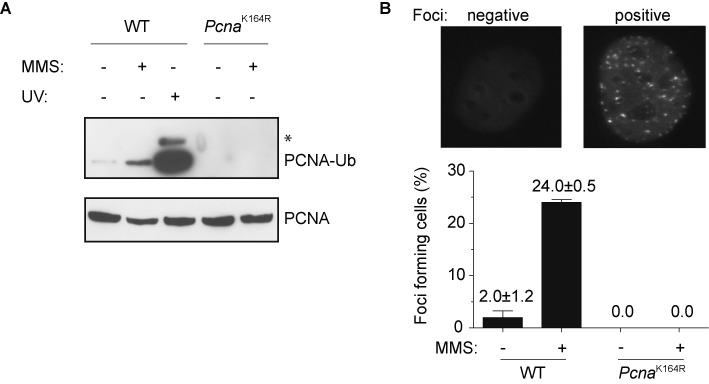
eGFP-Polκ foci formation after MMS treatment. (**A**) Detection of PCNA-Ub after MMS treatment by immunoblotting. Chromatin-bound proteins were isolated from WT or *Pcna*^K164R^ cells (MEFs) that were mock treated or continuously exposed to MMS (0.75 mM) for 6 h and separated with SDS-PAGE. After blotting, the membrane was cut below the 38 kDa marker and subsequent antibody staining and ECL was performed separately. The part of the membrane containing proteins with molecular weights >38 kDa (containing PCNA-Ub) was exposed for the maximum time, while the lower part of the membrane (<38 kDa proteins, containing unconjugated PCNA) was exposed only shortly. The asterisk indicates PCNA modified with two ubiquitin moieties. A representative experiment is shown. (**B**) WT and *Pcna*^K164R^ MEFs containing eGFP-Polκ were continuously exposed to MMS (0.75 mM) for 6 h after which they were fixed in 4% paraformaldehyde and analyzed with fluorescent microscopy. The average of three independent experiments is shown ±SD.

Several TLS polymerases display subnuclear focus formation following exposure to agents that stall replication forks. We next asked whether Polκ can form distinct subnuclear foci independently of PCNA-Ub, after MMS exposure. Compared to UV irradiation, MMS treatment weakly induces PCNA-Ub in WT, but not in *Pcna*^K164R^ cells (Figure 2A). The relative weak induction of mammalian PCNA-Ub has been observed in previous studies (45,46). Remarkably, MMS failed to induce eGFP-Polκ foci in *Pcna*^K164R^ cells, whereas under the same conditions about 25% of WT cells formed foci (Figure 2B). This result indicates that, following MMS treatment, Polκ focus formation completely depends on PCNA-Ub.

### Replication fork progression after MMS treatment

**Figure 3.**
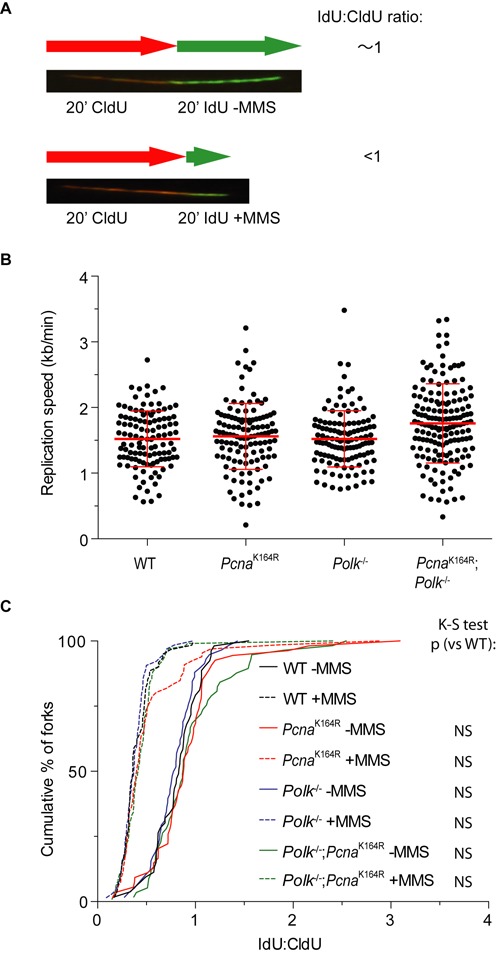
Replication fork progression analysis after MMS treatment. (**A**) Scheme of DNA fiber analysis experiment. MEFs were first labeled with 25 μM CldU for 20 min and then 20 min with 250 μM IdU, or 250 μM IdU and 3 mM MMS. The length of CldU and IdU tracks was measured and the average replication speed (kb/min, error bars represent SD) on a non-damaged template of the CldU track was calculated (**B**). (**C**) Analysis of IdU:CldU ratios of mock and MMS-treated WT, *Polk*^−/−^, *Pcna*^K164R^ and *Polk*^−/−^; *Pcna*^K164R^ MEFs. Data are presented as cumulative percentage of forks at each ratio. At least 50 DNA fibers were analyzed per experiment. A representative experiment is shown (of 2). The Kolmogorov–Smirnov (K-S) test was performed to determine statistical significance. NS means not significant. None of the tested genotypes show a significant difference (K-S test, p > 0.05) compared to wild-type cells.

**Figure 4.**
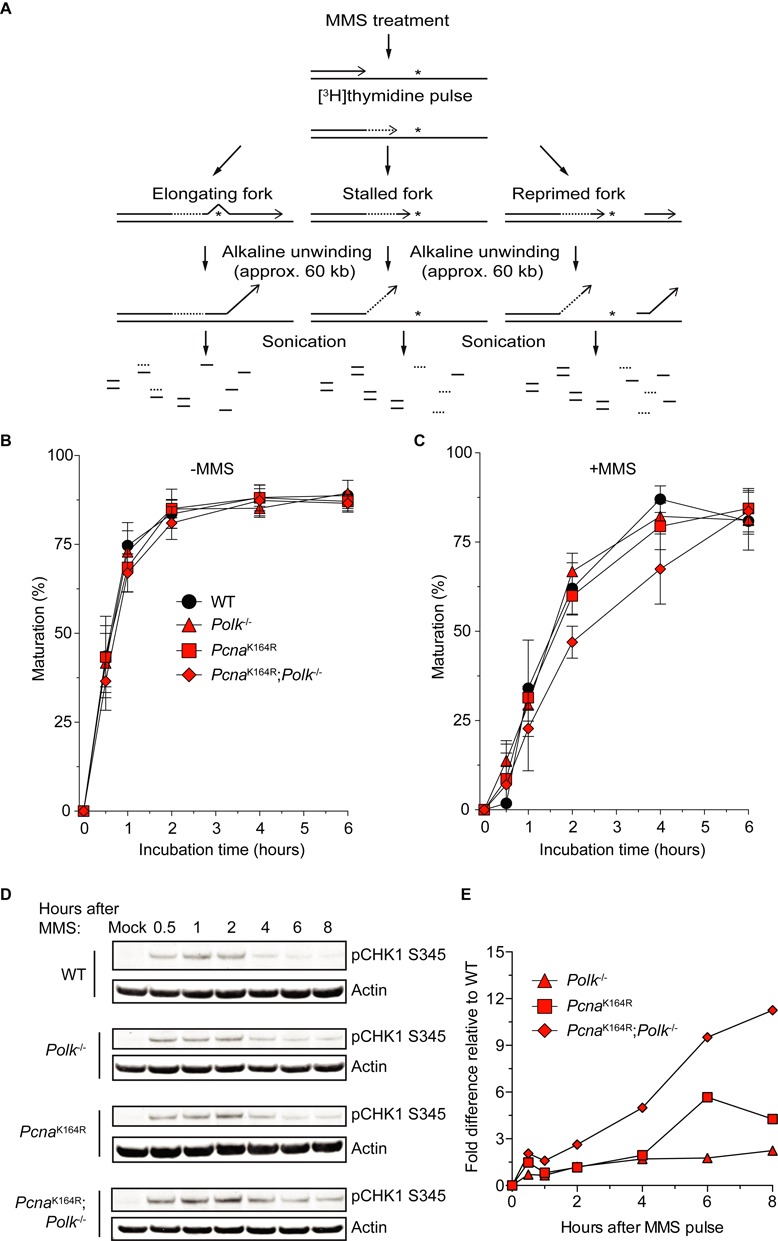
Replication block recovery and ATR/Chk1 activation after MMS treatment of *Pcna*^K164R^; *Polk*^−/-^ MEFs. (**A**) Scheme of ADU experiment. First, cells are treated with 1.5 mM MMS for 30 min.The asterisks represent MMS-induced replication fork stalling DNA lesions. Then, cells are pulse labeled with [^3^H]thymidine and chased for different time points as indicated. Subsequently, free DNA ends are locally unwound by alkaline treatment, after which the DNA is sheared by sonication. After binding to hydroxylapatite, ssDNA and dsDNA are eluted separately using appropriate potassium phosphate buffers. Finally, radioactivity is measured in the fractions corresponding to either ssDNA or dsDNA. Adapted from (49). (**B**) mock-treated MEFs. (**C**) MMS-treated cells. Average of five independent experiments ±SD. (**D**) Detection of activated CHK1. p53 knock down immortalized MEFs were treated for 30 min with 5 mM MMS after which they were harvested after indicated time points. Whole cell lysates were analyzed by immunoblotting with a pCHK1^S345^-specific antibody. (**E**) Quantification of immunoblot data from (D). All data are relative to WT and normalized to actin levels. Average of two independent experiments.

Given the specific role for Polκ and the critical contribution of PCNA-Ub in the survival of MMS, we here determined whether Polκ depends on PCNA-Ub during MMS-induced DDT using DNA fiber analyses and alkaline DNA unwinding assays (ADU). The DNA fiber analysis allows visualization of individual replicons on a single molecule level. It cannot however detect close-coupled repriming events, as these gaps are too small to be detected by the DNA fiber assay. The ADU assay determines the persistence of radioactively labeled single stranded DNA ends of elongating replication forks and thereby measures both on the fly and post-replicative bypass events. To exclude a function of Polκ or PCNA^K164^ during unperturbed replication, we first determined the replication speed prior to MMS treatment using the DNA fiber analysis. As expected (Figure 3A and B), there was no significant difference between the four genotypes, indicating that PCNA^K164^ modification and Polκ do not control unperturbed replication. Next, we analyzed on the fly DNA damage bypass in the presence of MMS (Figure 3A and C). Irrespective of the genotype, the presence of MMS resulted in a lower IdU:CldU ratio, indicative of replication fork stalling. Apparently, on the fly TLS of MMS-induced lesions was not affected by loss of PCNA-Ub and/or Polκ (Figure 3C). To investigate the relevance for PCNA-Ub and/or Polκ in TLS in post-replicative DNA damage bypass of MMS-induced lesions, we applied ADU in cells exposed to MMS or after mock treatment. As shown in Figure 4B, untreated cells of all four genotypes displayed a similar, time-dependent, increase in the amount of matured replicons, which suggests that replication fork progression at undamaged DNA is independent of PCNA-Ub or Polκ, confirming our DNA fiber analysis data. When treated with MMS, all cell lines displayed delayed replication fork progression as compared with untreated cells, indicating that, according to our experimental setup, not all MMS-induced DNA damage was repaired by base excision repair (BER) and thus plentiful to still cause replication fork stalling (Figure 4A) (47), although we cannot rule out a role for PCNA-Ub and Polκ during BER of MMS-induced DNA damage. Moreover, MMS-treated *Pcna^K164R^;Polk^−/−^* cells showed a delayed replication fork progression as compared to WT cells, whereas fork progression was marginally, but not significantly, delayed in the single mutant cells (Figure 4C). Taken together, although these results do not rule out the involvement of Polκ and PCNA-Ub in a common pathway, they show that in addition, they can act in a different, redundant pathway to bypass MMS-induced DNA damage.

### ATR/Chk1 activation after MMS treatment

Delayed replication fork progression may lead to accumulation of ssDNA, either at the stalled fork or by the generation of post-replicative gaps (1). Persistent ssDNA activates the Ataxia telangiectasia and Rad3 related (ATR)/Chk1 DNA damage checkpoint (48). To test whether loss of PCNA-Ub or Polκ resulted in increased checkpoint activation we determined levels of phosphorylated Chk1 (pChk1) by immunoblotting. After MMS treatment, increased levels of pChk1 that persisted over a longer period were found in the *Pcna*^K164R^ and Polκ single mutant cells as compared with WT cells (Figure 4D and E). These effects were exacerbated in the double mutant cells (Figure 4D and E), indicative for enhanced accumulation of ssDNA regions and activation of the ATR/Chk1 DNA damage checkpoint in these cells.

### Replication stress and S-phase progression after MMS treatment

**Figure 5.**
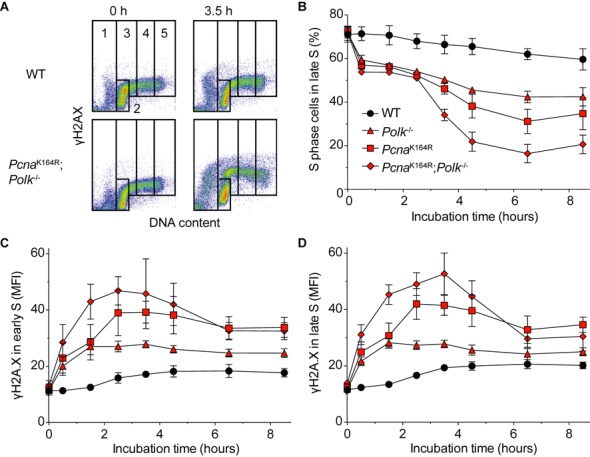
γH2A.X formation and cell cycle progression of *Pcna*^K164R^; *Polk*^−/−^ pre-B cells. (**A**) Examples of the gating strategy. The gates 1–5 are indicated to determine the frequency of cells in sub G1 (1), G1 (2), early S (3), late S (4) and G2 (5). Adapted from (38). (**B**) 2 × 10^5^ pre-B cells per condition were pulse treated with 100 μM MMS for 30 min at 37°C and fixed after the pulse at indicated time points. DNA content was visualized by TO-PRO®-3 staining and measured by FACS. (**C**) As panel (A), but analysis of γH2A.X in the same experiment in early S-phase B cells. (**D**) As panel (B), but late S-phase cells. Average of two independent experiments with two independent cell lines per genotype *in duplo* is plotted ±SD.

The reduced replication fork progression and the activation of the ATR/Chk1 DNA damage checkpoint observed in *Pcna^K164R^;Polk^−/−^* cells exposed to MMS might result in a decrease in S phase progression and increased levels of replication stress. To test this hypothesis, we analyzed cell cycle progression and γH2A.X formation in S phase pre-B cells after pulse treatment with MMS (Figure 5). Indeed, cell cycle progression of *Pcna^K164R^;Polk^−/−^* cells was most affected by MMS treatment; a gradual decrease in the S-phase progression was observed with regard to the other genotypes: WT > *Polk*^−/−^ > *Pcna*^K164R^ > *Pcna^K164R^;Polk^−/−^* cells (Figure 5B). Consequently, a similar decrease is observed for cells that accumulate in the G2 phase (Supplementary Figure S1C). Upon MMS treatment a gradual increase in the γH2A.X signal, indicative for DNA strand discontinuities, was observed, as well as accumulation of sub-G1 cells G1: WT < *Polk*^−/−^ < *Pcna*^K164R^ < *Pcna^K164R^;Polk^−/−^* cells (Figure 5C and D and Supplementary Figure S1A).

### Replication fork progression and cell survival after MMS treatment of *Polh*^−/−^; *Polk*^−/−^ mutant cells

**Figure 6.**
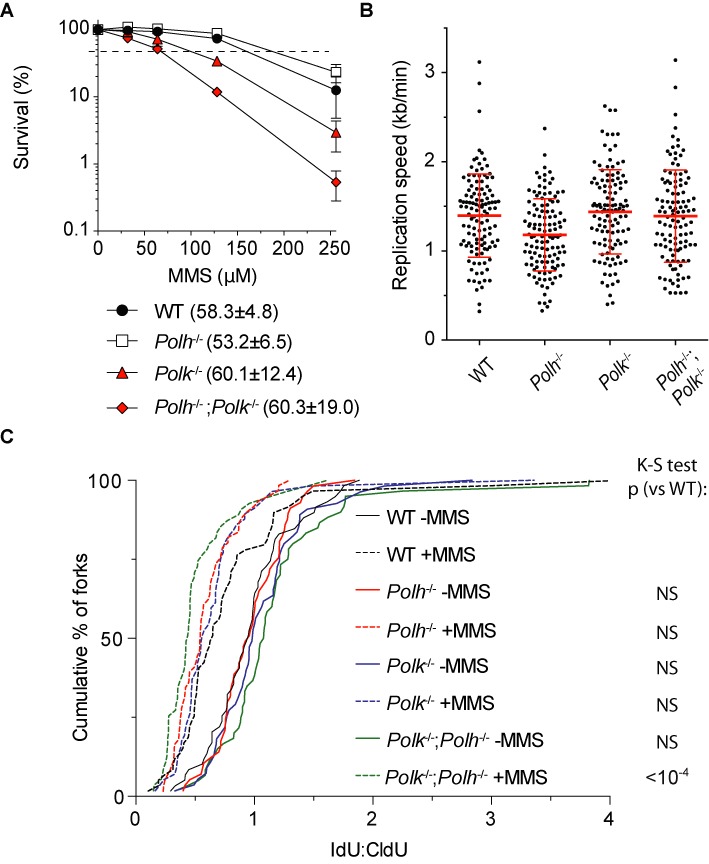
Colony survival and replication fork analysis of *Polh*^−/−^; *Polk*^−/−^ MEFs. (**A**) Colony survival after MMS treatment of SV40 immortalized WT, *Polh*^−/−^, *Polk*^−/−^ and *Polh*^−/−^; *Polk*^−/-^ cells. Colony survival was normalized to the mock-treated cells for each condition and corrected for plating efficiency. Average of at least four independent experiments is plotted ±SD. The dashed line indicates 50% survival. Values behind the genotype description indicate average plating efficiencies ±SD. (**B**) Replication speed on non-damaged CldU track of SV40 immortalized WT, *Polh*^−/−^, *Polk*^−/−^ and *Polh*^−/−^; *Polk*^−/-^ cells. Average of at least 50 analyzed DNA fibers is shown ±SD. (**C**) Replication fork stalling analysis as in Figure 3C of SV40 immortalized WT, *Polh*^−/−^, *Polk*^−/−^ and *Polh*^−/−^; *Polk*^−/-^ cells. Data are presented as cumulative percentage of forks at each ratio. At least 50 DNA fibers were analyzed per experiment. The Kolmogorov–Smirnov (K-S) test was performed to determine statistical significance. NS means not significant. Only MMS-treated *Polh*^−/−^; *Polk*^−/−^ cells show a significant difference (K-S test, p < 10^−4^) compared to MMS-treated wild-type cells. A representative experiment (of 2) is shown.

**Figure 7.**
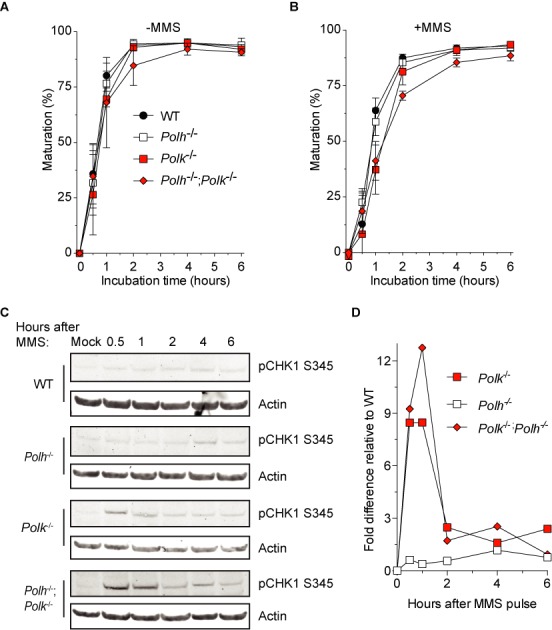
Replication block recovery and ATR/Chk1 activation after MMS treatment of *Polh*^−/−^; *Polk*^−/−^ MEFs. (**A,B**) Strand maturation as measured as in Figure 4A. Average of five independent experiments ±SD. (**C**) Detection of activated CHK1. SV40-immortalized MEFs were treated for 30 min with 5 mM MMS after which they were harvested after indicated time points. Whole cell lysates were analyzed by immunoblotting with a pCHK1^S345^-specific antibody. (**D**) Quantification of immunoblot data from (C). All data are relative to WT and normalized to actin levels. Average of two independent experiments.

Our observations, and those of others (49), suggest that Polη and Polκ are active during late DNA damage bypass. While this can be deduced by analyzing single mutant cell lines, we reasoned that due to the known redundancy among TLS polymerases, like between Polη and Polκ (44,50), defects in on the fly DNA damage bypass might not be observed in single TLS-deficient mutant cells. We therefore analyzed *Polh*^−/−^; *Polk*^−/−^ double knockout cells. First, we determined the sensitivity of WT, *Polh*^−/−^, *Polk*^−/−^ and *Polh*^−/−^; *Polk*^−/−^ cells to MMS. As revealed in Figure 6A, WT and *Polh*^−/−^ cells were equally sensitive to MMS treatment, which is consistent with previous reports describing Polη-deficient mouse pre-B cells (37) and Polη-deficient avian DT40 cells (51), and suggests that Polη is not the primary TLS polymerase to bypass MMS-induced DNA damage. Like Polκ-deficient pre-B cells (Figure 1), *Polk*^−/−^ MEFs were more sensitive to MMS treatment than WT and *Polh*^−/-^ cells. Interestingly, *Polh*^−/−^; *Polk*^−/−^ cells were even more sensitive than *Polk*^−/−^ cells, indicating that although Polη is not the primary choice for the bypass of MMS-induced lesions, in the absence of Polκ it is indeed involved in the bypass of this type of DNA damage. Next, we analyzed on the fly replication fork progression using the DNA fiber analysis. While unperturbed DNA replication occurred normally in *Polh*^−/−^; *Polk*^−/−^ cells (Figure 6B), replication fork stalling after MMS treatment was even more pronounced in these cells when compared to WT or the respective single mutants (Figure 6C). This difference was highly significant (p < 10^−4^, Kolmogorov–Smirnov test). This result indicates that Polη and Polκ are involved in ‘on the fly’ DNA damage bypass. To investigate the redundancy between Polη and Polκ in TLS in post-replicative DNA damage bypass of MMS-induced lesions, we applied ADU in cells exposed to MMS or after mock treatment (Figure 7A). Similar to the DNA fiber analysis, *Polh*^−/−^; *Polk*^−/−^ cells behaved similarly as WT and the respective single mutants when mock treated. After MMS treatment however, replicon maturation in *Polh*^−/−^; *Polk*^−/−^ cells was modestly, but significantly delayed compared to WT and *Polh*^−/-^ cells, while *Polk*^−/−^ cells were delayed only slightly (Figure 7B). We also analyzed ATR activation by measuring pChk1 levels in these cells. While WT and *Polh*^−/-^ cells hardly showed an induction of pChk1 levels after MMS treatment, *Polk*^−/−^ and *Polh*^−/−^; *Polk*^−/−^ cells did show increased pChk1 levels, with *Polh*^−/−^; *Polk*^−/−^ cells having the highest levels, indicative for enhanced accumulation of ssDNA regions and activation of the ATR/Chk1 DNA damage checkpoint in these cells (Figure 7C and D).

Taken together, both Polκ and modification of PCNA at K164 are important for efficient bypass of MMS-induced DNA damage, thereby preventing the accumulation of ssDNA and thus replication stress. Additionally, our data support the notion that Polκ can function independently of PCNA modification in a lesion-specific manner. We also showed that Polη can function as an important backup polymerase for Polκ during TLS of MMS-induced lesions.

## DISCUSSION

Having established a unique set of mammalian cell lines with defined deficiencies in DDT, we here investigated the dependence of Polκ on PCNA-Ub in the cellular response to various DNA damaging agents. We also investigated the role of Polη, Polκ and PCNA-Ub in coordinating the timing of TLS, i.e. on the fly and post-replicative DNA damage bypass. We found that cells deficient for Polκ and PCNA modification are more sensitive and accumulate more replication stress in response to MMS treatment than the respective single mutants. This indicates that Polκ can be activated in a PCNA-Ub-dependent and -independent manner. This feature, however, depends on the type of DNA damage, since Polκ completely depends on PCNA-Ub following exposure to BPDE.

The observed MMS sensitivity of Polκ-deficient pre-B cells is in line with previous studies in mouse embryonic stem cells and MEFs (19). Inactivation of *POLK* in *REV3*^−/−^ or *REV1*^−/−^ DT40 cells further sensitizes these cells to MMS (18,19). This observation suggests that Polκ can play a role in bypassing MMS-induced DNA lesions also in avian cells, albeit under conditions where the main players, Rev1 and Polζ, are absent. Thus, while in DT40 cells Polκ seems to serve as a backup TLS polymerase for the Rev1/Polζ pathway, in the mammalian system Polκ has a primary role in bypassing alkylated DNA bases. Interestingly, cells deficient for both Polη and Polκ show increased MMS sensitivity compared to cells that lack only Polκ, while single Polη-deficient cells do not display MMS sensitivity at all. Therefore, we conclude that Polη can act as a backup polymerase in the absence of Polκ during bypass of MMS-induced lesions. Interestingly, analogous observations have previously been made in a pro-mutagenic system, where Polκ can act as a backup polymerase of Polη during somatic hypermutation, but only in the complete absence of Polη (44,50). Furthermore, Polk has an important backup role in Polη-deficient *XPV* cells in response to UV light (52,53). These observations suggest that at a stalled fork, the preference for a specific TLS polymerase is highly lesion-specific.

MMS induces a plethora of DNA lesions, among which 3-methyladenine (3meA) and 7-methylguanine (7meG) are the most abundant (54). What specific lesions are bypassed by PCNA-Ub and Polκ-dependent TLS after MMS treatment? In contrast to 7meG, 3meA poses a replication block to replicative polymerases, but not to Y-family TLS polymerases (54,55). Polη, Polκ and Polι are all able to incorporate a nucleotide opposite, or extend from, a 3meA analog in *in vitro* primer extension assays, with Polκ being the most accurate (55). *In vivo* this might account for the sensitivity of the *Pcna^K^*^164R^ mutant cells, as PCNA-Ub is needed for efficient activation of at least Polκ in response to MMS-induced DNA damage. However, both 3meA and 7meG are prone to depurination, thereby leaving a highly mutagenic abasic site (54,55). Although conflicting data exist (56), almost all TLS polymerases are able to bypass abasic sites under defined *in vitro* conditions (11,57–59). Taken together, lesions that cause replication fork stalling after MMS treatment might be persistent 3meA and the abasic sites that are generated due to chemically instable 3meA and 7meG.

Based on a recent study proposing a role for PCNA-Ub and Polη in MSH2/MSH6-dependent repair synthesis in human cells in G_1_ phase after treatment with the oxidizing compound H_2_O_2_ (60), we tested whether cells deficient for Polκ, PCNA modification (*Pcna*^K164R^), or both, display sensitivity to H_2_O_2_. We found that in mouse cells all tested genotypes displayed the same sensitivity, suggesting no role of PCNA ubiquitination in the repair of H_2_O_2_-induced DNA damage. Future investigations should verify the relevance of this discrepancy.

Our data indicated that MMS-induced Polκ focus formation completely depends on PCNA-Ub, as no Polκ foci were observed in *Pcna*^K164R^ cells. Importantly, the percentage of cells that displayed Polκ focus formation is relatively low. This observation is in accordance with Ogi *et al.*, who have shown that a higher percentage of cells shows focus formation of Polη and PCNA compared to Polκ in response to UV light (61). Given the additional finding that Polκ can be activated in a PCNA-Ub-independent manner, our data on MMS-induced Polκ foci formation further support the notion that focus formation is not a prerequisite for TLS activation, as is also demonstrated by the fact that a Polη PIP mutant does not rescue UV-induced focus formation, but does restore UV resistance (62,63).

We found that post-replicative DNA damage bypass of MMS-induced lesions partly relies on both Polκ and PCNA-Ub, as shown by ADU. Additionally, Polη and Polκ seem to play a redundant role in the late bypass as well. In the absence of these factors, replication stress accumulates to a higher extent and persists longer as compared to WT or Polκ-deficient cells. In addition to their role in the late bypass, Polη and Polκ also appear to be involved in DNA damage bypass on the fly, as shown by DNA fiber analysis. This model of the timing of TLS contrasts a model proposed by Elvers *et al.* suggesting that in human fibroblasts, Polη is primarily involved in post-replicative gap filling (i.e. late DNA damage bypass) after UV irradiation. Our explanation for this discrepancy is that apparently in the absence of only Polη, another TLS polymerase, perhaps Polκ, takes over its function. Consequently, no major defects in on the fly DNA damage bypass are measured in the DNA fiber assay using cells that lack only one particular TLS polymerase.

In summary, we have shown that in response to MMS treatment PCNA-Ub-dependent and PCNA-Ub-independent pathways can activate Polκ, a finding similar to Polη activation in response to UV-induced lesions (37). It remains to be investigated what factors control Polκ in the absence of PCNA-Ub. Potential candidates are unmodified PCNA, the alternative DNA clamp RAD9-RAD1-HUS1, shown in fission yeast to physically interact with Polκ (64), Rev1 (24) or yet unknown regulators of TLS. Additionally, we have provided evidence for a backup role of Polη in the TLS response to MMS-induced lesion in the absence of Polκ. These results indicate that the choice for a particular TLS polymerase is lesion dependent. How a replication fork activates the right TLS polymerase for a specific lesion remains unclear and is currently under investigation.

## SUPPLEMENTARY DATA

Supplementary Data are available at NAR Online.

SUPPLEMENTARY DATA
