# Supplementary material for: Roles of PCNA ubiquitination and TLS polymerases κ and η in the bypass of methyl methanesulfonate-induced DNA damage
Source: Nucleic Acids Res. 2014 Dec 10;43(1):282–94. doi: 10.1093/nar/gku1301 (PMC4288191; doi:10.1093/nar/gku1301)
Supplement: SUPPLEMENTARY DATA [file supp_43_1_282__index.html]

Roles of PCNA ubiquitination and TLS polymerases κ and η in the bypass of methyl methanesulfonate-induced DNA damage — SUPPLEMENTARY DATA 

# Roles of PCNA ubiquitination and TLS polymerases κ and η in the bypass of methyl methanesulfonate-induced DNA damage

## SUPPLEMENTARY DATA

**Files in this Data Supplement:**

- SUPPLEMENTARY DATA
