# Supplementary material for: Roles of PCNA ubiquitination and TLS polymerases κ and η in the bypass of methyl methanesulfonate-induced DNA damage
Source: Nucleic Acids Res. 2014 Dec 10;43(1):282–94. doi: 10.1093/nar/gku1301 (PMC4288191; doi:10.1093/nar/gku1301)
Supplement: SUPPLEMENTARY DATA [file supp_gku1301_nar-02445-d-2014-File009.pdf]

Supplemental Figure 1

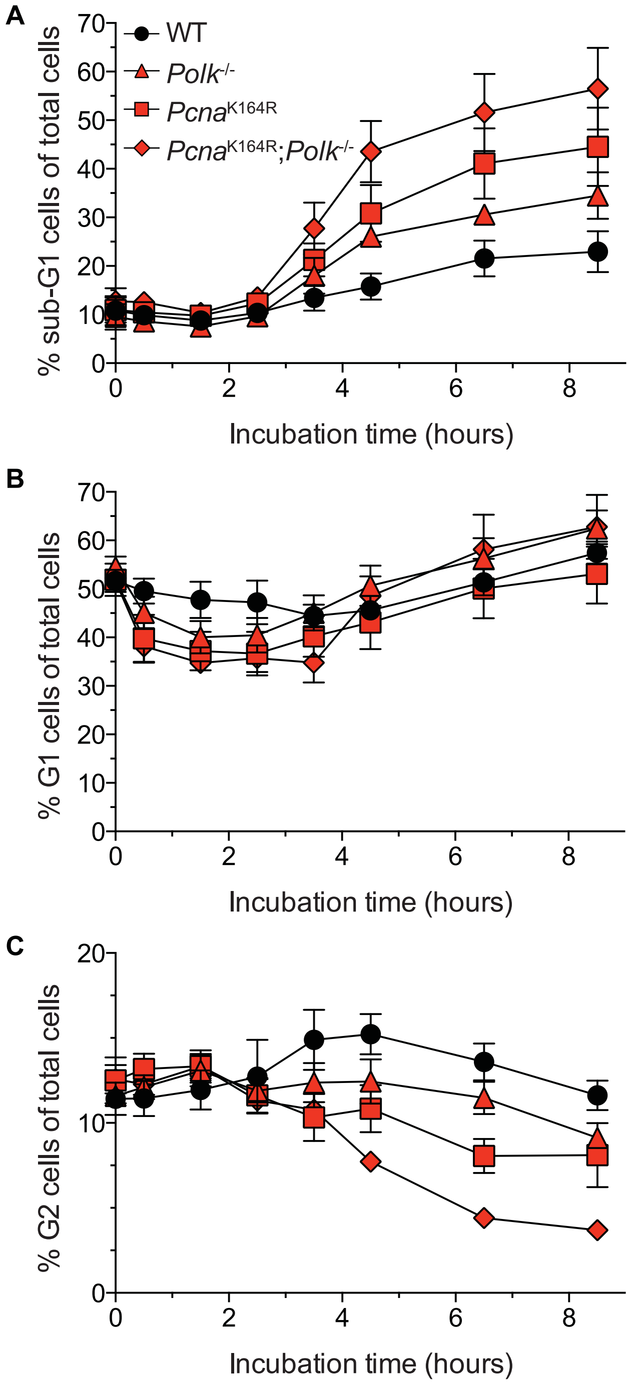

SUPPLEMENTAL FIGURE 1: Cell cycle progression of PcnaK164R;Polk-/- pre-B cells. As Fig. 5. A) Sub-G1 cells) B) G1 cells C) G2 cells.
